# Depletion of γδ T Cells Leads to Reduced Angiogenesis and Increased Infiltration of Inflammatory M1-like Macrophages in Ischemic Muscle Tissue

**DOI:** 10.3390/cells11091490

**Published:** 2022-04-29

**Authors:** Christoph Arnholdt, Konda Kumaraswami, Philipp Götz, Matthias Kübler, Manuel Lasch, Elisabeth Deindl

**Affiliations:** 1 Walter-Brendel-Centre of Experimental Medicine, University Hospital, Ludwig-Maximilians-Universität München, 81377 Munich, Germany; christoph.arnholdt@med.uni-muenchen.de (C.A.); kumaraswami.konda@med.uni-muenchen.de (K.K.); p.goetz@med.uni-muenchen.de (P.G.); matthias.kuebler@med.uni-muenchen.de (M.K.); manuel_lasch@gmx.de (M.L.); 2 Biomedical Center, Institute of Cardiovascular Physiology and Pathophysiology, Faculty of Medicine, Ludwig-Maximilians-Universität München, 82152 Planegg-Martinsried, Germany; 3 Department of Otorhinolaryngology, Head and Neck Surgery, University Hospital, Ludwig-Maximilians-Universität München, 81377 Munich, Germany

**Keywords:** angiogenesis, γδ T cells, gamma delta T cells, proliferation, macrophages, macrophage polarization, neutrophils, neutrophil extracellular traps, NETs, ischemia

## Abstract

γδ T cells, a small subset of T cells in blood, play a substantial role in influencing immunoregulatory and inflammatory processes. The functional impact of γδ T cells on angiogenesis in ischemic muscle tissue has never been reported and is the topic of the present work. Femoral artery ligation (FAL) was used to induce angiogenesis in the lower leg of γδ T cell depleted mice and wildtype and isotype antibody-treated control groups. Gastrocnemius muscle tissue was harvested 3 and 7 days after FAL and assessed using (immuno-)histological analyses. Hematoxylin and Eosin staining showed an increased area of tissue damage in γδ T cell depleted mice 7 days after FAL. Impaired angiogenesis was demonstrated by lower capillary to muscle fiber ratio and decreased number of proliferating endothelial cells (CD31^+^/BrdU^+^). γδ T cell depleted mice showed an increased number of total leukocytes (CD45^+^), neutrophils (MPO^+^) and neutrophil extracellular traps (NETs) (MPO^+^/CitH3^+^), without changes in the neutrophils to NETs ratio. Moreover, the depletion resulted in a higher macrophage count (DAPI/CD68^+^) caused by an increase in inflammatory M1-like macrophages (CD68^+^/MRC1^−^). Altogether, we show that depletion of γδ T cells leads to increased accumulation of leukocytes and M1-like macrophages, along with impaired angiogenesis.

## 1. Introduction

The occlusion of arterial vessels, being the main cause of the most prevalent forms of cardiovascular diseases (CVD), namely coronary heart disease (CHD), cerebrovascular accidents (CVA) and peripheral artery diseases (PAD), can often only be treated by invasive medical methods, which always present a certain risk for the patient. CVD in general have been the leading cause of death for years [1] and belong to the severe pathologies identified in SARS-CoV-2 patients [2,3]. Despite some improvements in the prevention and treatment of CHD and PAD, these diseases still represent a substantial medical burden worldwide [4]. The reduced perfusion in the affected tissue caused by the narrowing or complete occlusion of a vessel leads—without immediate treatment—to insufficient supply of oxygen and nutrients in the peripheral tissue, and after some time to ischemic tissue damage [5]. Ischemia in turn works as a stimulus to increase capillarity. This process is called angiogenesis [6,7] and can be explained by the development of new capillaries from the pre-existing vasculature, either by sprouting or by splitting; the most studied variant of angiogenesis is sprouting angiogenesis. Here, hypoxia leads to an increased release of vascular endothelial growth factor (VEGF-A), followed by a cascade which ultimately causes endothelial cells (ECs) to proliferate into the surrounding matrix and form solid sprouts [6]. Splitting angiogenesis, on the other hand, also known as intussusceptive angiogenesis, represents a process of reorganization and a strong increase in the capillary network by splitting existing vessels into two [6,7,8]. Unregulated angiogenesis, however, can represent the onset of cancer and various ischemic and inflammatory diseases [9]. The balance of various pro- and anti-angiogenic cytokines and enzymes, the influence of different cell types (such as ECs and pericytes and their interaction) and the components of the extracellular matrix regulate the growth and remodeling of capillary networks. Hereby, the supply of oxygen and nutrients can be ensured [10].

Under stimulation of various influencing factors, such as fMet-Leu-Phe (fMLP) or tumor necrosis factor α (TNF-α), neutrophils can release a large amount of VEGF-A and thus make a crucial contribution to angiogenic growth by enhancing endothelial cell (EC) proliferation [11,12,13]. In addition, several studies have shown a positive angiogenic effect through neutrophil extracellular traps (NETs) [14,15]. However, the enhancement of angiogenesis depends on a sensitive balance of NET formation, since excessive accumulation of NETs may in turn lead to cytotoxic damage to ECs and impaired tissue repair [16,17]. Macrophages also play a fundamental role in the angiogenic cascade by secreting growth factors, cytokines and enzymes, and facilitating neovascularization by modifying the extracellular matrix [18,19,20,21]. In addition, the polarization of macrophages was detected to have a crucial impact on angiogenesis: tissue containing predominantly anti-inflammatory and regenerative macrophages of the M2 phenotype showed enhanced angiogenesis, whereas an accumulation of pro-inflammatory M1-like macrophages led to a deteriorating angiogenic process [22,23].

B and T lymphocytes represent, among many other cell types, the cellular components of the immune system, which are also particularly essential elements of the adaptive immune system. In addition to their role as actors in the immune defense, it has been shown that different subgroups of B and T lymphocytes have a critical role in regulating angiogenesis as well [24,25,26,27]. With only 3–5% of all CD3^+^ cells and compared to αβ T cells, γδ T cells present only a small fraction of all T lymphocytes in peripheral blood [28]. The difference between these two subgroups lies in the different structure of the T cell receptor, which consists in the αβ T cells of an alpha and a beta chain, whereas a gamma and a delta chain form the receptor of TCRγδ^+^ T cells. In contrast to αβ T cells, γδ T cells do not contain CD4 or CD8 co-receptors; thus, antigen recognition is not restricted to MHC molecules [29]. However, γδ T cells interact with a broad range of different antigens, such as small peptides, proteins, phospholipids or sulfatides. γδ T cells, natural killer (NK) cells and other cell types express the receptor natural killer group 2 member D (NKG2D). This receptor can bind MHC class 1-related ligands, such as MHC class I chain-related protein A and B (MICA and MICB) and UL16-binding protein (ULBP) [30], whose expression can be induced by stress, transformation of cells and DNA damage, and thus activate γδ T cells [31,32,33,34,35].

These data indicate that the depletion of γδ T cells might have an influencing effect on the recruitment of leukocytes, polarization of macrophages and angiogenesis in ischemic muscle tissue. Interestingly, the direct impact of γδ T cells on sterile, ischemia-induced angiogenesis has never been investigated and is the subject of the present study.

## 2. Materials and Methods

### 2.1. Animal Protocol and Treatments

The following procedures were all performed after approval from the Bavarian Animal Care and Use Committee (ethical approval code: ROB-55.2Vet-2532.Vet_02-17-99) in strict accordance with German and NIH animal welfare and legislation guidelines. To investigate the role of γδ T cells, 8–10 week old C57BL/6J (Charles River Laboratories, Sulzfeld, Germany) mice with γδ T cell depletion were compared to wildtype C57BL/6J mice without any treatment and—as negative control to exclude unrecognized side effects caused by the depleting antibody—to C57BL/6J mice treated with an isotype control antibody (ISO). For γδ T cell depletion, a single dose of 200–250 µg of anti-γδ TCR mAb clone UC7-13D5 (cat. no. 107517, BioLegend, San Diego, CA, USA) was injected intravenously (i.v.) one day before the surgical intervention. The control group was treated with the same concentration of Ultra-LEAF™ Purified Armenian Hamster IgG isotype control antibody clone HTK888 (cat. no. 400959, BioLegend). All mice were administered 1.25 mg bromodeoxyuridine (BrdU, dissolved in phosphate-buffered saline (PBS)) (Sigma-Aldrich, St. Louis, MO, USA) i.p. daily, beginning directly after surgery.

### 2.2. Experimental Procedures and Tissue Harvesting

Before the surgery, a combination of anesthetics consisting of midazolam (5.0 mg/kg, Ratiopharm GmbH, Ulm, Germany), fentanyl (0.05 mg/kg, CuraMED Pharma, Karlsruhe, Germany) and medetomidine (0.5 mg/kg, Pfister Pharma, Berlin, Germany) were injected subcutaneously. After anesthetizing, unilateral ligation of the right femoral artery was performed, while the same operation was done on the left side without closing the surgical thread to obtain an internal sham control [5]. The operation led to unilateral initiation of angiogenesis in the gastrocnemius muscle, which could be investigated by further tissue processing. Tissue collection was performed on day 3 or day 7 after ligation of the femoral artery. For this purpose, after another anesthesia, the hindlimb was perfused first with adenosine buffer (5% bovine serum albumin (BSA, Sigma-Aldrich) and 1% adenosine (Sigma-Aldrich, dissolved in PBS)) and for fixation of the muscle tissue with 3% paraformaldehyde (PFA, Merck, Darmstadt, Germany; dissolved in PBS, pH 7.4). Finally, gastrocnemius muscle from both legs was harvested and stored at −80 °C after being embedded in Tissue-Tek compound (Sakura Finetek Germany GmbH, Staufen, Germany).

### 2.3. Histology and Immunohistology

The cryopreserved tissue blocks of the gastrocnemius muscle were cut into 8–10 μm thick slices. Gastrocnemius muscle sections of day 3 aFAL were used for neutrophil and NETs staining, whereas ECs, leukocytes, macrophages, activated VEGF receptor 2 (Tyr1175) and the extent of ischemic tissue damage were analyzed in tissue samples isolated 7 days aFAL.

To investigate the area of ischemic muscle tissue out of the total gastrocnemius muscle on day 7 aFAL, Hematoxylin and Eosin (H&E) staining was conducted.

Endothelial cells were stained along with BrdU and leukocytes (CD45). Therefore, cryo-sections were incubated with pre-warmed 1N HCL at 37 °C for 30 min in a humid chamber. The tissue was then permeabilized using 0.2% Triton X-100 solution (AppliChem GmbH, Darmstadt, Germany; 10 min at room temperature (RT)) in 1 × PBS/0.5% BSA/0.1% Tween-20 (AppliChem GmbH) and blocked with 10% goat serum (Abcam, cat. ab7481, Cambridge, UK; dissolved in 1 × PBS/0.5% BSA/0.1% Tween-20) for 1 h at RT. BrdU staining was now performed using the primary antibody mRat BrdU (Abcam, ab6326; diluted 1:50 in blocking solution; incubated at 4 °C overnight) and the secondary antibody GantiRat Alexa Fluor 546 (Thermo Fisher Scientific, A11081, Waltham, MA, USA; diluted 1:100 in PBST; incubated at RT for 1 h). After secondary blocking with 1 × PBS/4% BSA/0.1% Tween-20 (for 30 min at RT), endothelial cells were stained using the antibody Ranti-mouse CD31-Alexa Fluor^®^ 647 (BioLegend, 102516, diluted 1:50 in 1 × PBS/0.1% Tween-20 (PBS-T); for 2 h at RT), and leukocytes were stained using anti-CD45-Alexa Fluor^®^ 488 antibody (BioLegend, 11-0451-85, diluted 1:100 in PBS-T for 2 h at RT).

To characterize angiogenesis by VEGF receptor 2 activation at tyrosine 1175 in addition to quantification of capillaries in relation to the number of muscle fibers and Brdu^+^ ECs per muscle fiber, co-staining of CD31 and phospho-VEGF receptor 2 at tyrosine 1175 (19A10) rabbit mAb (Cell Signaling Technology, 2478, Danvers, MA, USA; dilution 1:100 in PBS) was performed. Donkey anti-rabbit IgG Alexa Fluor^®^ 488 antibody (Thermo Fisher Scientific, A-21206, dilution 1:200 in PBS) served as a secondary antibody for the phospho-VEGFR-2 (Tyr1175) antibody. All remaining staining steps were analogous to the CD31/CD45/BrdU staining.

To study neutrophils and NETs on muscle tissue collected 3 days aFAL, fixation was performed with 4% PFA (for 10 min at RT), permeabilization with 0.2% Triton X-100 (dissolved in 1 × PBS/0.5% BSA/0.1% Tween-20 for 2 min at RT) and blocking with 10% donkey serum (Abcam, ab7475; dissolved in 1 × PBS/0.5% BSA/0.1% Tween-20 for 1 h at RT). Subsequently, incubation with primary antibodies anti-citrullinated histone H3 (Cit-H3; polyclonal rabbit anti-Histone H3 (citrulline R2 + R8 + R17), Abcam, ab5103, diluted 1:100 in blocking solution) and anti-myeloperoxidase (MPO; R&D Systems, AF3667, Minneapolis, MN, USA; diluted 1:20 in blocking solution) at 4 °C overnight and incubation with secondary antibodies donkey anti-goat Alexa Fluor 594 (Thermo Fisher Scientific, A-11058, diluted 1:100 in PBS-T) and donkey anti-rabbit Alexa Fluor 488 antibody (Thermo Fisher Scientific, A-21206, diluted 1:200 in PBS-T) for 1 h at RT was performed.

To investigate macrophages and their polarization, sections were fixed (4% PFA, 5 min at RT), blocked (4% BSA, 1 h at RT) and incubated with the primary antibody anti-MRC1 (mannose receptor C-type 1; Abcam, ab64693, diluted 1:200 in PBS; incubation overnight at 4 °C) and secondary antibody donkey anti-rabbit IgG Alexa Fluor 546 (Thermo Fisher Scientific, A-10040, dilution 1:200 in PBS-T; incubation for 1 h at RT). Additionally, incubation with anti-CD68 Alexa Fluor 488 antibody (Abcam, ab201844, diluted 1:200 in PBS-T) overnight at 4 °C was performed.

For more detailed classification of macrophage populations into pro- and anti-inflammatory macrophages, sections were permeabilized (0.2% Triton X-100 in PBS; 10 min at RT) and incubated with either purified anti-mouse IL-10 antibody (BioLegend, 5050001, dilution 1:50 in PBS) or TNF-α monoclonal antibody (MP6-XT22, Thermo Fisher Scientific, 14-7321-81, diluted 1:100 in PBS) overnight at 4 °C. For IL-10, as well as TNF-α, secondary antibody donkey anti-rat IgG Alexa Fluor Plus 647 (Thermo Fisher Scientific, A48272, diluted 1:200 in PBS; incubation for 1 h at RT) was used.

Additionally, to label nucleic DNA, all sections were counter-stained with DAPI (Thermo Fisher Scientific, 62248, diluted 1:1000 in PBS; incubation for 10 min at RT) and mounted with Dako mounting medium (Agilent, Santa Clara, CA, USA).

Using the 20× objective (415 μm × 415 μm) of a confocal LSM 880 (Carl-Zeiss Jena GmbH, Jena, Germany) and the 20× objective (630 μm × 475 μm) of an epifluorescence microscope (Leica DM6 B, Leica microsystems, Wetzlar, Germany), stained gastrocnemius muscle tissue was analyzed. To comparatively analyze the CD31/CD45/BrdU/DAPI, CD31/Phospho-VEGF receptor 2 (Tyr1175) and CD68/MRC1/TNF-α or IL-10/DAPI stains of the 3 groups (wildtype, isotype control, and TCR γ/δ depletion), epifluorescence microscope was used. For this purpose, we selected 5 defined areas of ischemic muscle tissue to count cells. To evaluate angiogenesis, we first counted all CD45^+^/DAPI^+^ cells (leukocytes) and then inferred CD31^+^/CD45^−^ cells as ECs. To analyze proliferating ECs, we also checked for colocalization with an intranuclear positive BrdU signal. Macrophages (CD68^+^ cells) were first counted and then differentiated by their polarization using the anti-MRC1 antibody. H&E stainings were also imaged using the epifluorescence microscope. For neutrophils and NETs quantification, 5 confocal pictures were analyzed.

To obtain a negative control of our immunohistochemical staining and to guarantee a valid assessment of the immunofluorescence images, the primary antibody was omitted for unconjugated antibodies (BrdU, MRC1, MPO, CitH3, phospho-VEGF receptor 2 (Tyr1175), IL-10, TNF-α), whereas the sham-operated muscles were comparatively assessed for conjugated antibodies (CD31, CD45, CD68, DAPI).

For all immunohistochemical studies, gastrocnemius muscles of 5 mice per group were evaluated. In addition, for all quantitative studies, we evaluated 5 images from ischemic regions of the gastrocnemius muscle per mouse per group.

The open-source program ImageJ (Wayne Rasband, retired from National Institutes of Health) and ZEN 3.2 software (blue edition, Carl Zeiss AG, Wetzlar, Germany) were used for cell counting and measurements of the percentage of tissue.

### 2.4. Statistical Analyses

Statistical analyses and graph plottings were performed using GraphPad Prism 8 (GraphPad Software, La Jolla, CA, USA). All data are stated as means ± standard error of the mean (SEM). Statistically significant results were considered at *p* ≤ 0.05.

### 2.5. Graphical Abstract

The graphical abstract was created with Biorender.com (accessed on 24 April 2022).

## 3. Results

To investigate the influence of γδ T lymphocytes on angiogenesis in ischemic muscle tissue, we used the well-established murine hindlimb model of ischemia [5]. For that purpose, the right femoral artery was ligated in a surgical procedure, while the left leg was sham operated. This led to unilateral reduced perfusion of the lower leg, resulting in ischemia in the gastrocnemius muscle. All investigations were performed comparatively between wildtype (WT), isotype antibody-treated (ISO), and γδ T cell depleted (TCRγδ depl.) mice.

### 3.1. γδ T Cell Depleted Mice Show Increased Ischemic Tissue Damage

**Figure 1:**
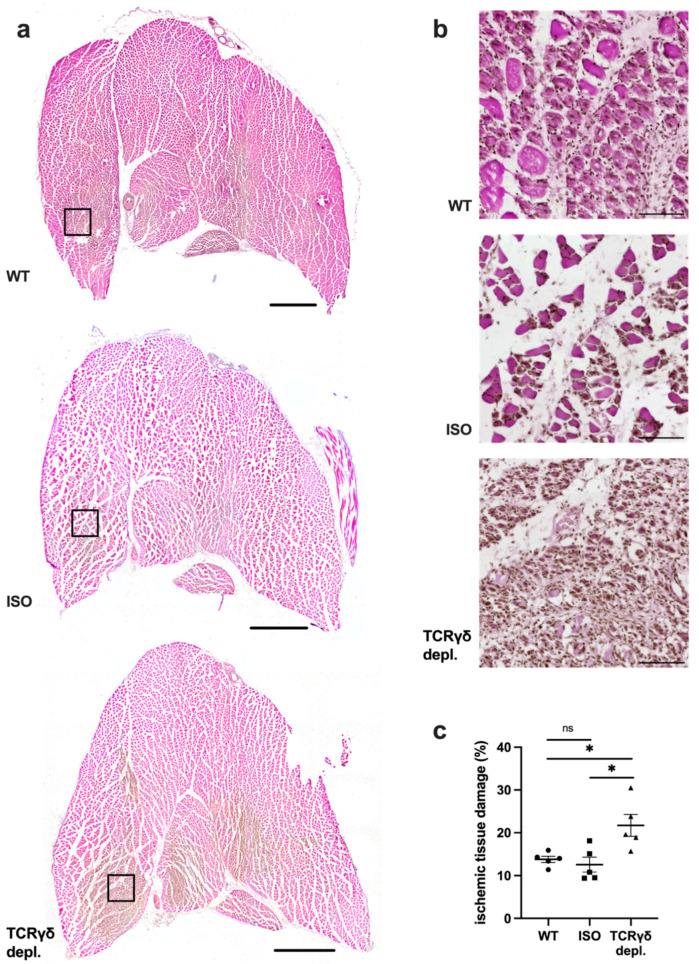
γδ T cell depleted mice show increased ischemic tissue damage. (**a**) Representative H&E pictures of gastrocnemius muscles of wildtype (WT) (top), isotype antibody-treated (ISO) (middle) and γδ T cell depleted mice (bottom) 7 days after femoral artery ligation (aFAL). Scale bars: 1000 µm. (**b**) Detailed images of the black boxes shown in (**a**). Scale bars: 100 µm. (**c**) The scatter plot shows the area of ischemic tissue damage (%) of WT, ISO and TCRγδ T cell depleted mice 7 days aFAL. Data are means ± SEM, *n* = 5 per group. * *p* < 0.05, ns ≥ 0.05 (WT vs. ISO vs. TCRγδ depletion) by one-way ANOVA with the Tukey’s multiple comparisons test.

To evaluate and compare the tissue damage of WT, ISO and γδ T cell depleted mice, H&E stains were performed on gastrocnemius muscles collected on day 7 after femoral artery ligation. As expected, no ischemic damage was found in samples of sham-operated legs in any group of mice (data not shown). In the gastrocnemius muscles of the occluded side, ischemic damage was found in all muscles (Figure 1a,b), with a significantly increased area of ischemic tissue damage in the γδ T cell depleted group (Figure 1c), while gastrocnemius muscles of the ISO and the WT group showed an almost similar extent of tissue damage.

### 3.2. γδ T Cell Depletion Leads to Reduced Capillarity

**Figure 2:**
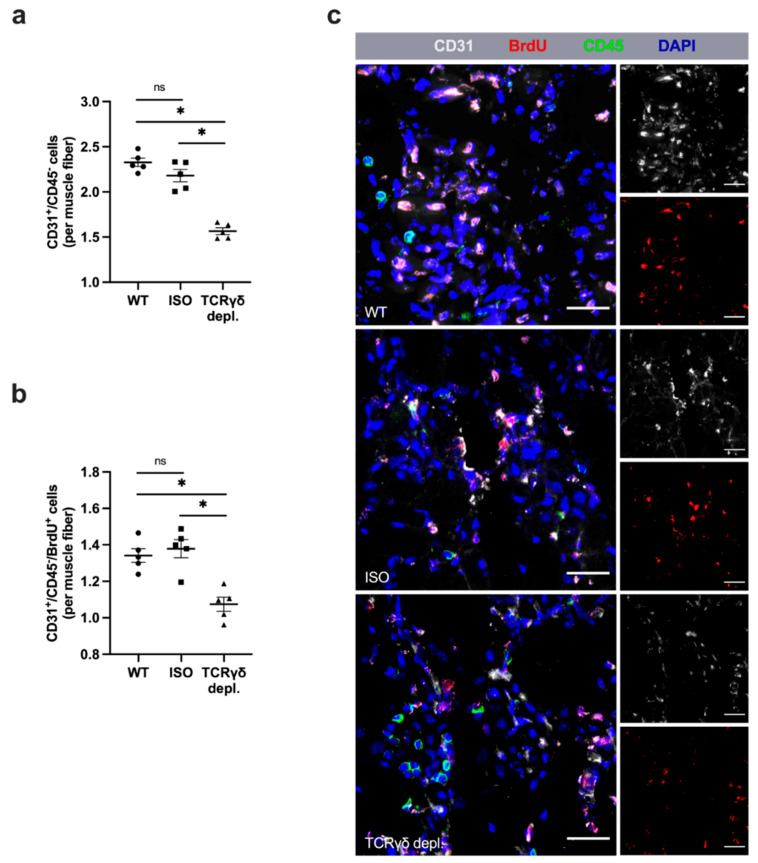
Absence of γδ T cells decreases angiogenesis. Scatter plots show the number of (**a**) endothelial cells (CD31^+^/CD45^−^) and (**b**) proliferating endothelial cells (CD31^+^/CD45^−^/BrdU^+^) per muscle fiber of ischemic gastrocnemius muscles of WT, ISO and γδ T cell depleted mice 7 days after femoral artery ligation (aFAL). Data are means ± SEM, *n* = 5 per group. * *p* < 0.05, ns ≥ 0.05 (WT vs. ISO vs. TCRγδ T cell depletion) by one-way ANOVA with the Tukey’s multiple comparisons test. (**c**) Representative images of ischemic gastrocnemius muscles of WT (top), isotype antibody-treated (middle) and TCRγδ T cell depleted mice (bottom) 7 days aFAL. Single channel pictures (small images) show endothelial cells (CD31, gray) and proliferating cells (BrdU, red). Merged images also show leukocytes (CD45, green) and nuclei (DAPI, blue). Scale bars: 30 µm.

To investigate the specific influence of γδ T cells on angiogenesis, the capillary to muscle fiber ratio of ischemic muscle tissue collected 7 days aFAL was analyzed using CD31/CD45/BrdU/DAPI staining. CD31 antibody was used as an endothelial cell marker, CD45 as a panleukocyte marker, BrdU in combination with CD31 as a marker for proliferating ECs, and DAPI to stain nuclei. To exclude CD31^+^ leukocytes, only CD31^+^/CD45^−^/DAPI^+^ cells were counted as ECs. γδ T cell depleted mice showed a decreased capillary to muscle fiber ratio compared to WT and ISO mice (Figure 2a,c). In addition, a decreased ratio of proliferating ECs per muscle fiber was observed in γδ T cell depleted mice (Figure 2b,c). WT and ISO mice showed both a similar capillary to muscle fiber ratio and a similar ratio of proliferating ECs per muscle fiber (2 a,b,c). The non-ischemic muscles isolated from sham-operated mice in all three groups showed no significant differences in capillarity or in the ratio of proliferating ECs per muscle fiber (data not shown).

### 3.3. γδ T Cell Depletion Leads to Reduced Activation of VEGF Receptor 2 (Tyr1175)

**Figure 3:**
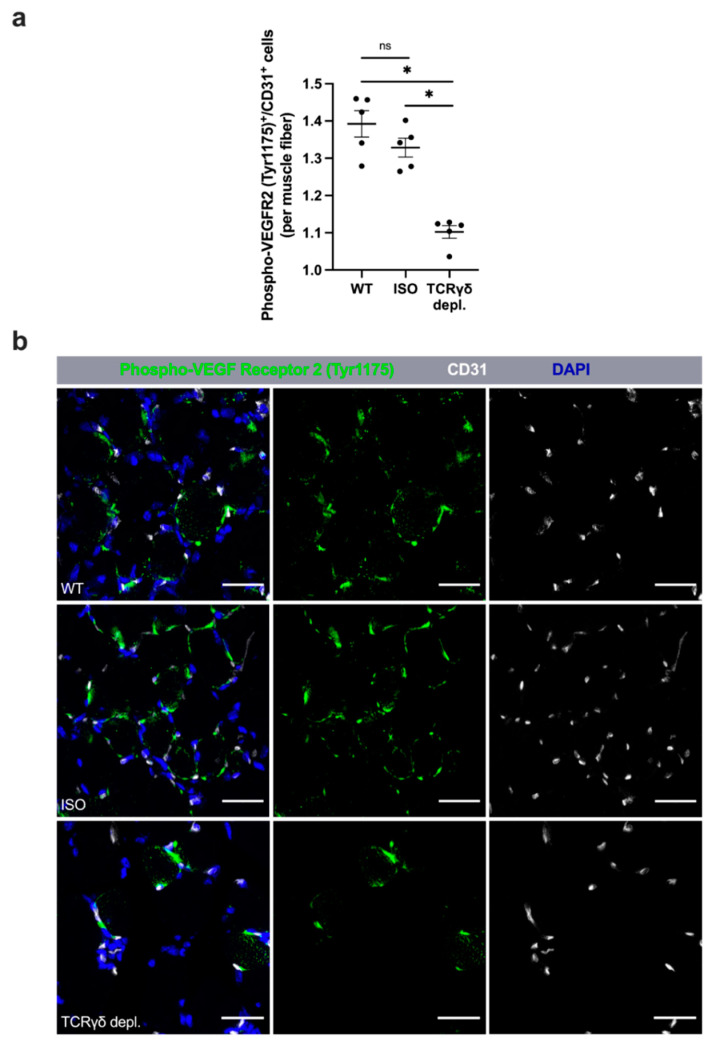
γδ T cell depletion leads to reduced activation of VEGF receptor 2 (Tyr1175). (**a**) The scatter plot shows the number of VEGF receptor 2 (Tyr1175)/CD31 double-positive endothelial cells (Phospho-VEGF receptor 2 (Tyr1175)^+^/CD31^+^) per muscle fiber in ischemic gastrocnemius muscles of WT, ISO and TCRγδ T cell depleted mice 3 days after femoral artery ligation (aFAL). Data are means ± SEM, *n* = 5 per group. * *p* < 0.05, ns ≥ 0.05 (WT vs. ISO vs. TCRγδ depletion) by one-way ANOVA with the Tukey’s multiple comparisons test. (**b**) Representative images of ischemic gastrocnemius muscles of wildtype (WT, top), isotype antibody-treated (ISO, middle) and TCRγδ T cell depleted mice (TCRγδ depl., bottom) 3 days aFAL. The VEGF receptor 2 was stained with an antibody recognizing the activated phospho-VEGF receptor 2 (Tyr1175) form (green). Endothelial cells were stained with an antibody against CD31 (white), while nuclei were labeled using DAPI (blue). Scale bars: 30 µm.

To examine the influence of γδ T cells on the phosphorylation of VEGF receptor 2 at tyrosine 1175, and thus its activation, muscle tissue collected on day 3 aFAL was triple stained for phospho-VEGF receptor 2 (Tyr1175), CD31 and DAPI. γδ T cell depleted mice showed significantly decreased activated VEGF receptor 2 (Tyr1175) compared to WT and ISO mice (Figure 3a,b). In nonischemic muscle tissue of sham-operated mice, no significant difference in the number of activated VEGF receptor 2 (Tyr1175) was found in all three groups (data not shown).

### 3.4. γδ T Cell Depleted Mice Show Enhanced Leukocyte Infiltration

**Figure 4:**
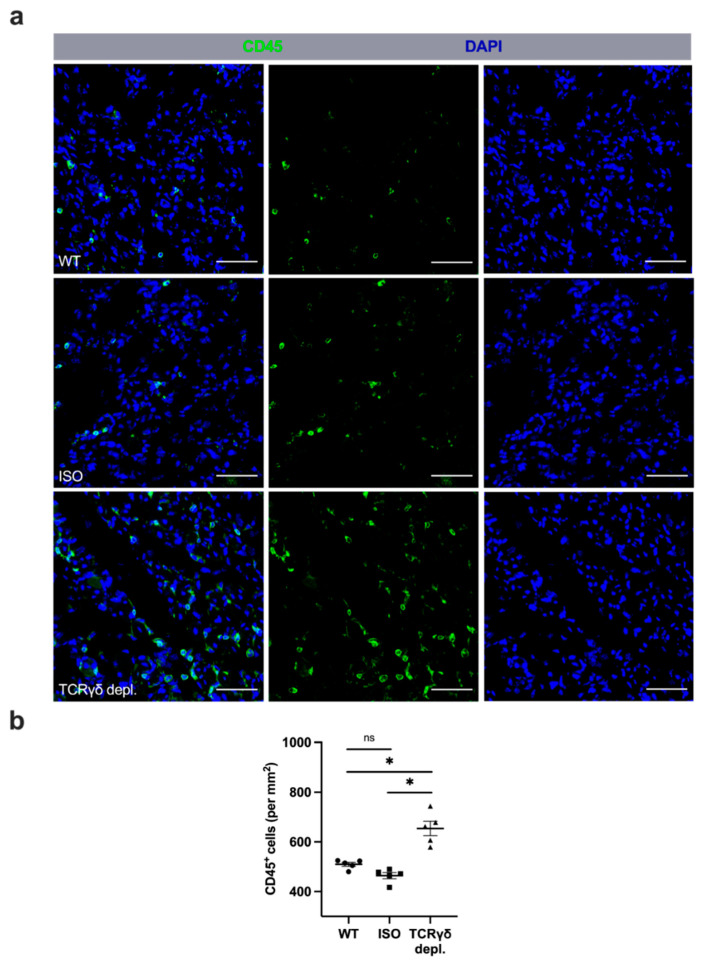
Mice lacking γδ T cells showed increased accumulation of leukocytes in ischemic muscle tissue. (**a**) Representative immunofluorescence images of analyzed gastrocnemius muscles of WT (top), isotype antibody-treated (middle) and TCR γδ T cell depleted mice (bottom) 7 days after femoral artery ligation (aFAL). Leukocytes were stained with an antibody against CD45 (green), while nuclei were labeled using DAPI (blue). Scale bars: 50 µm. (**b**) The scatter plot shows the absolute number of leukocytes (CD45^+^) per mm^2^ in ischemic gastrocnemius muscle tissue from WT, ISO and TCR γδ T cell depleted mice 7 days aFAL. Data shown are means ± SEM, *n* = 5 per group, a defined area of 1.5 mm^2^ of ischemic muscle was analyzed per mouse. * *p* < 0.05, ns ≥ 0.05 (WT vs. ISO vs. TCR γδ depletion) by one-way ANOVA with the Tukey’s multiple comparisons test.

To investigate the infiltration of leukocytes in ischemic tissue, we analyzed the tissue obtained on day 7 aFAL using the pan-leukocyte marker CD45. The ischemic area of the γδ T cell depleted group showed a significantly increased number of CD45^+^ leukocytes per mm^2^ and accordingly total number of leukocytes compared to the wildtype and isotype control groups (Figure 4a,b). Without initiation of ischemia (sham operation), there was no detectable difference in the number of CD45^+^ cells in the tissue among all three different treatment groups (Supplementary Materials, Figure S1).

### 3.5. γδ T Cell Depleted Mice Show Higher Amount of Neutrophils

**Figure 5:**
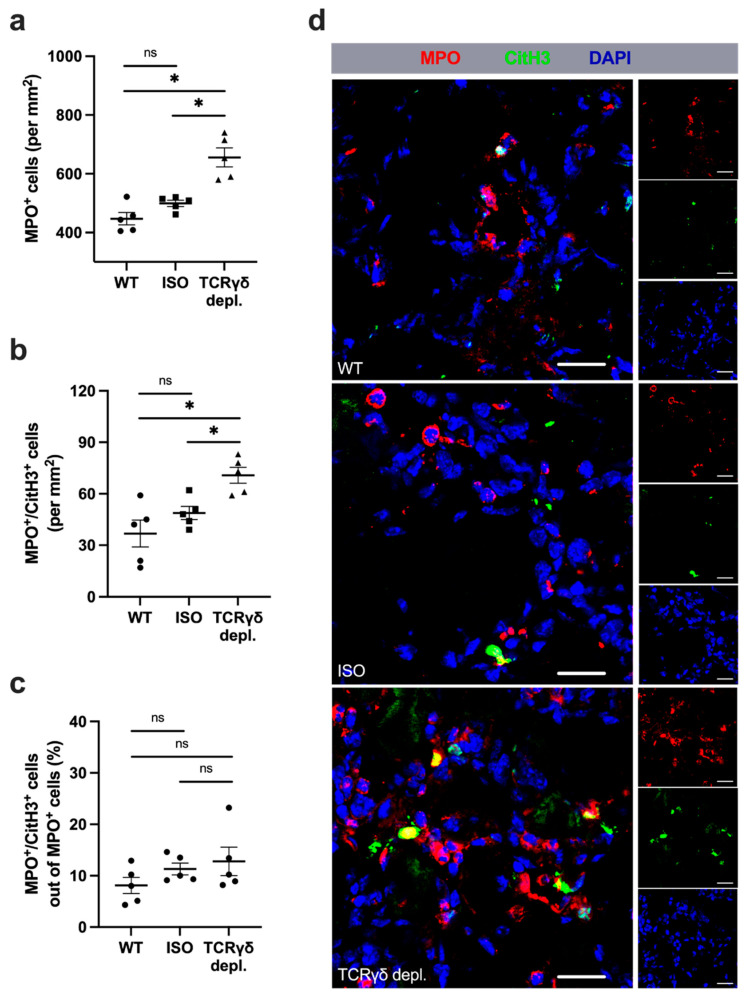
Absence of γδ T cells results in increased accumulation of neutrophils without affecting the formation of neutrophil extracellular traps. The scatter plots show (**a**) the total number of neutrophils (myeloperoxidase; MPO^+^ cells) per mm^2^ (upper plot), (**b**) the occurrence of NETs (MPO^+^/CitH3^+^ (citrullinated histone 3)) per mm^2^ (middle plot), and (**c**) the relative proportion of NETs positive MPO^+^ cells of all MPO^+^ cells (lower plot) in the ischemic gastrocnemius muscle tissue of mice of the WT, isotype antibody-treated and γδ T cell depleted group 3 days after femoral artery ligation (aFAL). Data are means ± SEM, *n* = 5 per group. * *p* < 0.05, ns ≥ 0.05 (WT vs. ISO vs. TCR γδ depletion) by one-way ANOVA with the Tukey’s multiple comparisons test. A defined area of 1.5 mm^2^ of ischemic gastrocnemius muscle tissue was analyzed per mouse. (**d**) Representative immunofluorescence images of ischemic gastrocnemius muscles from WT (top), ISO (middle) and TCR γδ T cell depleted mice (bottom) 3 days aFAL. Images show neutrophils (MPO^+^/DAPI^+^), NETs (MPO^+^/CitH3^+^/DAPI^+^) and nuclei (DAPI^+^) labeled with anti-MPO (red), anti-CitH3 (green) and DAPI (blue). Scale bars: 20 µm.

In order to assess the total number of neutrophils and the formation of NETs, muscle tissue collected on day 3 aFAL was triple stained for myeloperoxidase (MPO), for citrullinated histone H3 (CitH3) as well as for DAPI. MPO^+^/DAPI^+^ cells were counted as neutrophils and MPO^+^/CitH3^+^/DAPI^+^ cells were classified as NETs. The evaluation showed a significantly increased number of neutrophils in muscle tissue of γδ T cell depleted mice compared to WT and isotype control antibody-treated mice (Figure 5a,d). In addition, the amount of NETs per mm^2^ was significantly increased in comparison to the control groups (Figure 5b,d). However, the percentage of NETs related to the total number of neutrophils showed no significant differences in all three groups (Figure 5c,d). In nonischemic muscles of sham-operated mice, almost no neutrophils or NETs could be found in all three groups (Supplementary Materials, Figure S2). Moreover, there was no difference in the NETs to neutrophil ratio (data not shown).

### 3.6. γδ T Cell Depletion Leads to Increased Number of Macrophages with Inflammatory M1-like Polarization

To analyze the number and polarization of recruited macrophages in the gastrocnemius muscle, tissue collected 7 days aFAL was stained for CD68 and the mannose receptor C-type 1 (MRC1) to differentiate between M1-like polarized macrophages and M2-like polarized macrophages. CD68^+^/MRC1^+^/DAPI^+^ signals were interpreted as M2-like polarized macrophages, whereas M1-like polarized macrophages were defined as MRC1-negative cells (CD68^+^/MRC1^−^/DAPI^+^).

**Figure 6:**
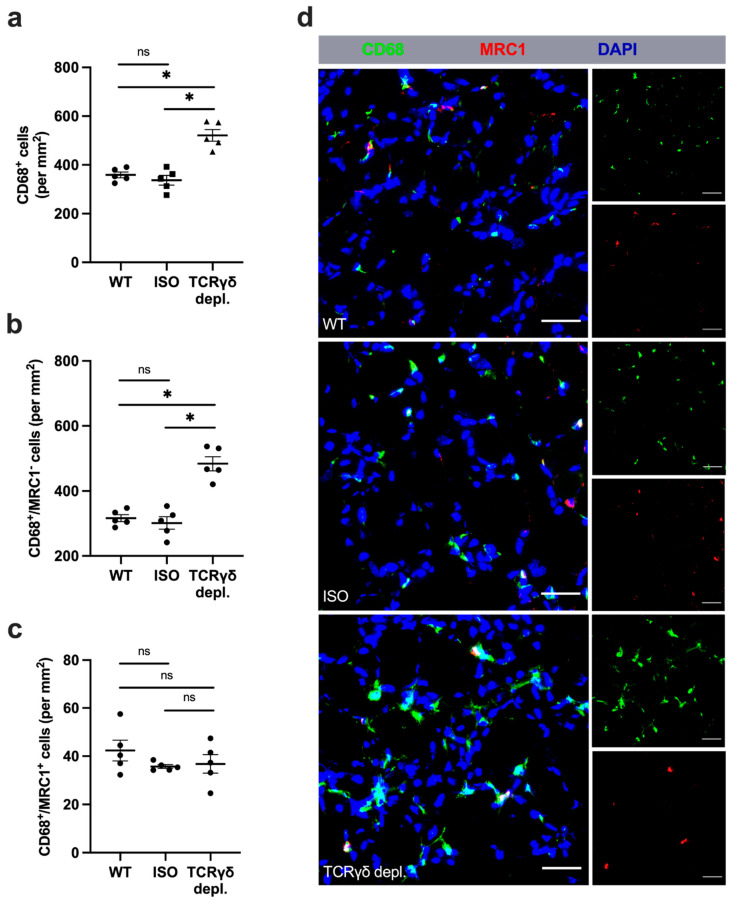
Depletion of γδ T cells leads to an increased number of M1-like polarized macrophages. Scatter plots show the absolute number of (**a**) all macrophages (CD68^+^) per mm^2^ (top plot), (**b**) MRC1-negative macrophages (CD68^+^/MRC1^−^ (mannose receptor C-type 1)) (middle plot) and (**c**) MRC1-positive macrophages (CD68^+^/MRC1^+^) (bottom plot) in ischemic gastrocnemius muscle tissue of wildtype (WT), isotype (ISO) and TCR γδ T cell depleted mice 7 days aFAL. Data are means ± SEM, *n* = 5 per group. * *p* < 0.05, ns ≥ 0.05 (WT vs. ISO vs. TCR γδ depletion) by one-way ANOVA with the Tukey’s multiple comparisons test. (**d**) Representative immunofluorescence images of analyzed ischemic muscle tissue of WT, ISO and TCR γδ T cell depleted mice. Macrophages (CD68^+^/DAPI^+^) were stained with antibodies targeting CD68 (green), MRC1 (red) and DAPI (blue). CD68^+^/MRC1^−^/DAPI^+^ cells were defined as M1-like polarized macrophages and CD68^+^/MRC1^+^/DAPI^+^ cells as M2-like polarized macrophages. Scale bars: 30 µm.

A significantly increased total number of macrophages was found in γδ T cell depleted mice in comparison to wildtype and isotype control antibody-treated mice (Figure 6a,d). Examination of macrophage polarization showed a significant increase in the absolute count of MRC1-negative macrophages (M1-like macrophages) in γδ T cell depleted mice compared to the two control groups, whereas no difference in the number of M2-like macrophages was found in all three groups (Figure 6b,c,d). To further investigate whether MRC1-negative macrophages (CD68^+^/MRC1^−^) show inflammatory features, co-staining of CD68 and MRC1, together with TNF-α as a pro-inflammatory marker, was performed (see Supplementary Materials, Figure S3). Indeed, our results evidenced that virtually all CD68^+^/MRC1^−^ macrophages expressed TNF-α and identified them as inflammatory macrophages. In contrast, all CD68^+^/MRC1^+^ macrophages expressed IL-10, identifying them as anti-inflammatory macrophages (see Supplementary Materials, Figure S4).

In non-ischemic muscles of sham-operated mice, no difference was found between all three groups either regarding the number of macrophages or their polarization (Supplementary Materials, Figure S5).

## 4. Discussion

In the present study, the influence of γδ T lymphocytes on the process of angiogenesis was studied. Our data demonstrated that the depletion of γδ T cells results in increased ischemic tissue damage and impaired angiogenesis. Increased infiltration of immune cells (CD45+ cells) was based on an increased number of neutrophils and macrophages. In addition, we found that the higher number of macrophages in γδ T cell depleted mice was due to a strong increase in inflammatory TNF-α^+^ M1-like polarized macrophages.

Neutrophils represent the first recruited cells of the innate immune system in our murine ischemic hindlimb model leading to inflammation and angiogenesis [36]. Their influence on angiogenesis is characterized by phagocytosis, removal of cell debris, participation in tissue repair and delivery of various growth factors [36]. Under hypoxic conditions, neutrophils play an important role in the initiation of angiogenesis by secreting VEGF-A and providing MMPs [37,38,39]. MMPs released by neutrophils, especially MMP-9, are responsible for the degradation of extracellular matrix components and through this mechanism are able to release further growth factors, including VEGF-A, which are bound to the extracellular matrix. The singularity of neutrophils among immune cells is their ability to release MMP-9 without the tissue inhibitor of metalloproteinases (TIMP), an endogenous inhibitor of MMP-9, and thus can positively influence angiogenesis [38]. However, our data, showing increased numbers of neutrophils with decreased angiogenesis in the γδ T cell depleted group, suggest that neutrophils may have had a negative effect on the angiogenic process. In addition to the pro-angiogenic properties of neutrophils, many reports have uncovered limiting effects on angiogenesis as well: In vitro studies have shown that upon proinflammatory stimulation, neutrophils can release proteolytically active elastase, which can cleave plasminogen to angiostatin fragments [40]. The resulting angiostatin fragments, comprising kringle domains 1–3, show an inhibitory effect on endothelial cell proliferation by degrading basic-fibroblast growth factor (bFGF) and VEGF [40,41]. Furthermore, the release of α-defensins by neutrophils can inhibit angiogenesis by preventing endothelial cell adhesion and blocking VEGF-induced endothelial cell proliferation [42]. In an ischemia-reperfusion injury (IRI) model in the lung, liver, kidney and intestine, Funken et al. showed that the absence of γδ T cells led to increased neutrophil recruitment, which is in line with our results [43]. The simultaneously higher tissue damage could also be a result of the release of reactive oxygen species (ROS) as neutrophils are known to release ROS, which may entail endothelial dysfunction and increased tissue damage [44].

Moreover, neutrophils have multiple strategies in the context of immune regulation, pathogen clearance and disease pathology. Phagocytosis, secretion of specialized granules and release of NETs are the three most important mechanisms to exert these tasks [45]. In particular, the formation of NETs and their impact on inflammatory processes has received increasing attention in recent years. Here, we evaluated by immunohistological analysis the neutrophil-derived formation of neutrophil extracellular traps (NETs) using MPO/CitH3 co-staining. We found an increased number of NETs, but no difference in the ratio of NETs to neutrophils. Hence, the depletion of γδ T cells does not influence the formation of NETs in our experimental setup. NETs are described as extracellular, web-like structures consisting of globular protein domains and DNA from neutrophils [46]. Regarding angiogenesis, different evidence was obtained: Aldabbous et al. demonstrated a positive angiogenic effect of NETs in studies of patients with pulmonary hypertension in vivo and in vitro [14]. However, a highly increased incidence of NETs did not seem to have any further beneficial effect on angiogenesis. Rather, a balance of NETs seems to be important for a positive angiogenic microenvironment [16]. Especially in wound healing, NET formation was not associated with improved angiogenesis, but with increased tissue damage [17,47]. Based on our data showing an increased occurrence of NETs caused by an increased number of neutrophils, we suggest that γδ T-cell depletion led to a pro-inflammatory NET formation that contributed to increased tissue damage and decreased angiogenesis.

Leukocytes with their manifold properties play a crucial role in the context of angiogenesis. The ischemic stimulus of damaged tissue resulting in inflammation leads to infiltration of leukocytes, whose transmigration to the tissue is facilitated by increased permeability of the endothelium [48]. Infiltrated leukocytes begin to remove cell debris, enhance local inflammation, and recruit additional immune cells, including neutrophils and macrophages. The release of pro-angiogenic factors, including VEGF-A [49], and proteins such as matrix metalloproteinase 9 (MMP9), responsible for remodeling of the extracellular matrix, contribute to further alteration of the microenvironment [23,36,50]. Rani et al. showed in a mouse model, studying the inflammatory process of acute lung injury (ALI) after trauma-hemorrhage (TH), that an absence of γδ T cells leads to an increase in inflammatory cells, such as monocytes or granulocytes [51]. These findings correspond to our results of increased infiltration of leukocytes, i.e., neutrophils and macrophages, and indicate that γδ T cells have a regulatory effect on the recruitment of different leukocyte subtypes. Another aspect that is in line with our findings of increased tissue damage is the fact that prolonged infiltration of leukocytes in ischemic tissue damage may also result in anti-angiogenic effects [50,52]. Several studies have shown that mice lacking γδ T cells displayed a prolonged inflammatory phase, whereas WT mice recovered faster from ischemia [53,54]. Furthermore, a rescue experiment showed that external administration of wildtype γδ T cells to the aforementioned mice led to resolution of inflammation and recovery [53].

Macrophages are well known as fundamental modulators of inflammation and angiogenesis. They represent an extremely plastic cell population due to their different phenotypes, which can affect angiogenesis, for example, through the release of paracrine-acting substances or directly as cellular chaperones that promote the fusion of vascular sprouts [48,55]. Depending on the prevailing stimuli, macrophages can polarize to pro-inflammatory M1 macrophages or to anti-inflammatory, regenerative M2 macrophages. However, the M1/M2 nomenclature is only valid for in vitro conditions and reflects the extreme edges of possible polarizations [56,57]. Accordingly, for in vivo situations, very often the terms M1-like and M2-like macrophages are used to indicate that these macrophages show pro-inflammatory features as M1 macrophages do, or anti-inflammatory, regenerative properties like M2 macrophages [58,59,60]. Accordingly, we denoted in our study CD68^+^/MRC1^−^/TNF-α^+^ macrophages as pro-inflammatory M1-like polarized macrophages and CD68^+^/MRC1^+^/IL-10^+^ macrophages as anti-inflammatory regenerative M2-like polarized macrophages.

By release of cytokines such as TNF-α or IL-10 and other paracrine signals, macrophages are considered to play an important role as enhancers of angiogenesis at the side of ischemic tissue [10,61,62,63]. At the onset of inflammation, M0-like monocytes mature into the pro-inflammatory M1-like macrophages with the function of phagocytosis, leukocyte recruitment and delivery of both pro-angiogenic factors (VEGF-A) and pro-inflammatory cytokines, such as TNF-α or IL-1β [23,61]. The pro-angiogenic influence of macrophages of the M1 phenotype is limited due to several reasons. One important reason is the matrix metalloproteinase-9 zymogen (proMMP-9) secreted by M1-like macrophages, which loses its pro-angiogenic effect through complexation with the tissue inhibitors of metalloproteinases metallopeptidase inhibitor 1 (TIMP-1) [64]. Polarization of macrophages to the M2 phenotype leads to downregulation of TIMP-1 expression, resulting in the release of TIMP-1 deficient, pro-angiogenic proMMP-9 [64]. Thus, the higher absolute number of M1-like macrophages in the tissue of γδ T cell depleted mice only leads to an increased level of TIMP-1 complexed pro-MMP-9, which cannot contribute to angiogenic processes. The pro-inflammatory phase is followed by the regenerative phase with the polarization of the initially classically activated macrophages (M1-like macrophages) to the alternatively activated M2-like macrophages [65,66]. M2-like macrophages can resolve the inflammatory conditions, provide tissue repair, and facilitate growth, tissue remodeling and thus angiogenesis [67,68]. Compared to WT and ISO mice, γδ T cell depleted mice showed in our experimental setup a strong predominance of pro-inflammatory M1-like macrophages along with an increased extent of tissue damage and reduced angiogenesis. Thus, the increased infiltration of macrophages (CD68^+^ cells) in γδ T cell depleted mice, which predominantly displayed M1-like polarization, may indicate an impaired switch to M2-like macrophages, which could be responsible for the reduced angiogenesis and the increased tissue damage. However, this is only an assumption, since mechanistic correlations are difficult to provide using in vivo experiments, and in vitro results do not always reflect the in vivo situation. Nevertheless, detailed investigations on the expression profile of the M1-like and M2-like macrophages should provide further insights and be topics of future examinations. Interestingly, a recent study has demonstrated that IFNγ priming of macrophages via epigenetic and transcriptional changes leads to decreased recruitment of leukocytes and therefore resolved inflammation [69]. Since T cells can secrete IFNγ, among others, it seems possible that the absence of IFNγ release from γδ T cells in the depleted mice could further account for the observed increase in leukocyte number.

Depletion of γδ T cells using the anti-γδ TCR mAb clone UC7-13D5 has been performed in many different experimental setups [70,71,72,73,74,75]. In addition to the depletion of γδ T cells, it is conceivable that side effects were induced by the administration of the antibody, which contribute to the observed findings in γδ T cell depleted mice. Indeed, it has been observed that treatment with the anti-γδ TCR mAb clone UC7-13D5 led to a slight and short-term increase in the percentage of αβ T lymphocytes, which, however, returned to normal levels within the first days [76]. Furthermore, changes in IFNγ production or in the activity of cytotoxic T cells and natural killer cells observed in other studies indicate that γδ T cell depletion may have led to similar side effects also in our experimental setting [77,78]. Finally, it is known that the binding of anti-γδ TCR mAb to γδ T cells leads to the formation of antigen-antibody complexes, resulting in the production and release of cytokines via activation of the complement system and further binding to Fcγ receptors on immune cells [79,80,81,82]. Accordingly, further detailed studies are necessary to define whether the observed effects (found in femoral artery ligated but not sham-operated legs) are exclusively due to the lack of γδ T cells or due to unrecognized side effects caused by the depleting antibody, although we did not find major differences between untreated and isotype control treated mice.

Ligation of the femoral artery results in reduced blood flow to the lower leg, leading to ischemia, local tissue damage and fibrosis [5,10]. It is known that increased tissue damage represents an increased ischemic stimulus for enhanced angiogenesis [83]. Our results showed that the increased tissue damage in the γδ T cell depleted group, however, was associated with reduced capillarity and therefore decreased angiogenesis. Thus, the depletion of γδ T cells did not result in improved angiogenesis compared to the WT and ISO groups despite the increased ischemic tissue damage. In addition to the initiation of angiogenesis in the lower leg, in our murine hindlimb model, ligation of the femoral artery simultaneously induces arteriogenesis in the thigh. Pre-existing collateral vessels, connecting the profunda femoral artery to the femoral artery, begin to grow (arteriogenesis) and are responsible for the additional blood supply to the lower leg. In 2016, Chillo et al. described that improved arteriogenesis results in decreased ischemic tissue damage in the lower leg [83]. Thus, an influence of a potentially comprised arteriogenesis on the degree of ischemic tissue damage found in the gastrocnemius muscle tissue of γδ T cell depleted mice cannot be excluded.

In general, tissue ischemia results in the development of new capillaries. Two mechanisms of angiogenesis are known: (a) sprouting angiogenesis, which is the formation of new capillary branches from pre-existing capillaries through the migration and proliferation of ECs, and (b) intussusception, in which splitting of a single capillary results in the formation of two vessels [6,7,8]. In our studies, we demonstrated that the depletion of γδ T cells led to reduced angiogenesis by a decreased number of proliferating ECs and a decreased capillary to muscle fiber ratio. The reduced number of proliferating ECs (BrdU^+^ ECs) indicates that sprouting angiogenesis was impaired in γδ T cell depleted mice. In addition, we found a reduced number of phospho-VEGFR-2 (Tyr1175) positive ECs in γδ T cell depleted mice. VEGFR-2 is considered the most important receptor for VEGF-A-mediated mitotic effects in ECs. Reduced activation of this receptor as reflected by reduced forms of VEGFR-2 phosphorylated at tyrosine 1175 points furthermore to reduced sprouting angiogenesis [84,85]. However, it also suggests that VEGF-A mediated mitotic signaling is impaired in γδ T cell depleted mice. However, intussusception, also referred to as splitting angiogenesis, might be affected as well. Dimova et al. showed in two experiments that mononuclear cells can contribute to the formation and stabilization of transluminal pillars, which is a prerequisite for the splitting of the preexisting vessels. Since lymphocytes belong to the mononuclear cells, it seems possible that the absence of γδ T cells in our experiment led to impaired intussusceptive angiogenesis. However, whether the mononuclear cells affecting splitting angiogenesis are actually monocytes or lymphocytes or other immune cells remains to be investigated [86,87].

In summary, we show that depletion of γδ T cells results in impaired angiogenesis and increased tissue damage in our murine hindlimb model of ischemia. Together with the increased infiltration of leukocytes, reflected by an increased number of neutrophils and predominantly pro-inflammatory M1-like polarized macrophages in γδ T cell depleted mice, our data indicate that γδ T cells play a crucial role in the context of angiogenesis and tissue regeneration.

## Data Availability

The data presented in this study are available on request from the first author.

## Supplementary Materials

The following supporting information can be downloaded at: https://www.mdpi.com/article/10.3390/cells11091490/s1, Figure S1: Representative immunofluorescence images of sham-operated legs showing low leukocyte count without any difference in number in WT, ISO and γδ T cell depleted mice; Figure S2: Representative immunofluorescence images of sham-operated legs showing almost no neutrophiles or neutrophil extracellular traps (NETs) in WT, ISO and γδ T cell depleted mice; Figure S3: MRC1 negative macrophages (CD68^+^/MRC1^−^) show co-staining with TNF-α; Figure S4: MRC1 positive macrophages (CD68^+^/MRC1^+^) show co-staining with IL-10; Figure S5: Representative immunofluorescence images of sham-operated legs showing low numbers of macrophages in WT, ISO and γδ T cell depleted mice.
