# Supplementary material for: Depletion of γδ T Cells Leads to Reduced Angiogenesis and Increased Infiltration of Inflammatory M1-like Macrophages in Ischemic Muscle Tissue
Source: Cells. 2022 Apr 29;11(9):1490. doi: 10.3390/cells11091490 (PMC9102774; doi:10.3390/cells11091490)
Supplement: Supplementary file 1 [file cells-11-01490-s001.zip › cells-1675309-supplementary.pdf]

cells

Article

Depletion of γδ T Cells Leads to Reduced Angiogenesis and Increased Infiltration of Inflammatory M1-like Macrophages in Ischemic Muscle Tissue

Christoph Arnholdt [1,2], Konda Kumaraswami [1,2], Philipp Götz [1,2], Matthias Kübler [1,2], Manuel Lasch [1,2,3] and Elisabeth Deindl [1,2,*]

1 Walter-Brendel-Centre of Experimental Medicine, University Hospital, Ludwig-Maximilians-Universität München, 81377 Munich, Germany; christoph.arnholdt@med.uni-muenchen.de (C.A.); kumaraswami.konda@med.uni-muenchen.de (K.K.); p.goetz@med.uni-muenchen.de (P.G.); matthias.kuebler@med.uni-muenchen.de (M.K.); manuel_lasch@gmx.de (M.L.)
2 Biomedical Center, Institute of Cardiovascular Physiology and Pathophysiology, Faculty of Medicine, Ludwig-Maximilians-Universität München, 82152 Planegg-Martinsried, Germany
3 Department of Otorhinolaryngology, Head and Neck Surgery, University Hospital, Ludwig-Maximilians-Universität München, 81377 Munich, Germany
* Correspondence: elisabeth.deindl@med.uni-muenchen.de; Tel.: +49-(0)-89-2180-76504

Citation: Arnholdt, C.; Kumaraswami, K.; Götz, P.; Kübler, M.; Lasch, M.; Deindl, E. Depletion of γδ T Cells Leads to Reduced Angiogenesis and Increased Infiltration of Inflammatory M1-like Macrophages in Ischemic Muscle Tissue. *Cells* **2022**, *11*, 1490. https://doi.org/10.3390/cells11091490

Academic Editor: Alessandro Poggi

Received: 25 March 2022
Accepted: 27 April 2022
Published: 29 April 2022

Publisher's Note: MDPI stays neutral with regard to jurisdictional claims in published maps and institutional affiliations.

Copyright: © 2022 by the authors. Submitted for possible open access publication under the terms and conditions of the Creative Commons Attribution (CC BY) license (https://creativecommons.org/licenses/by/4.0/).

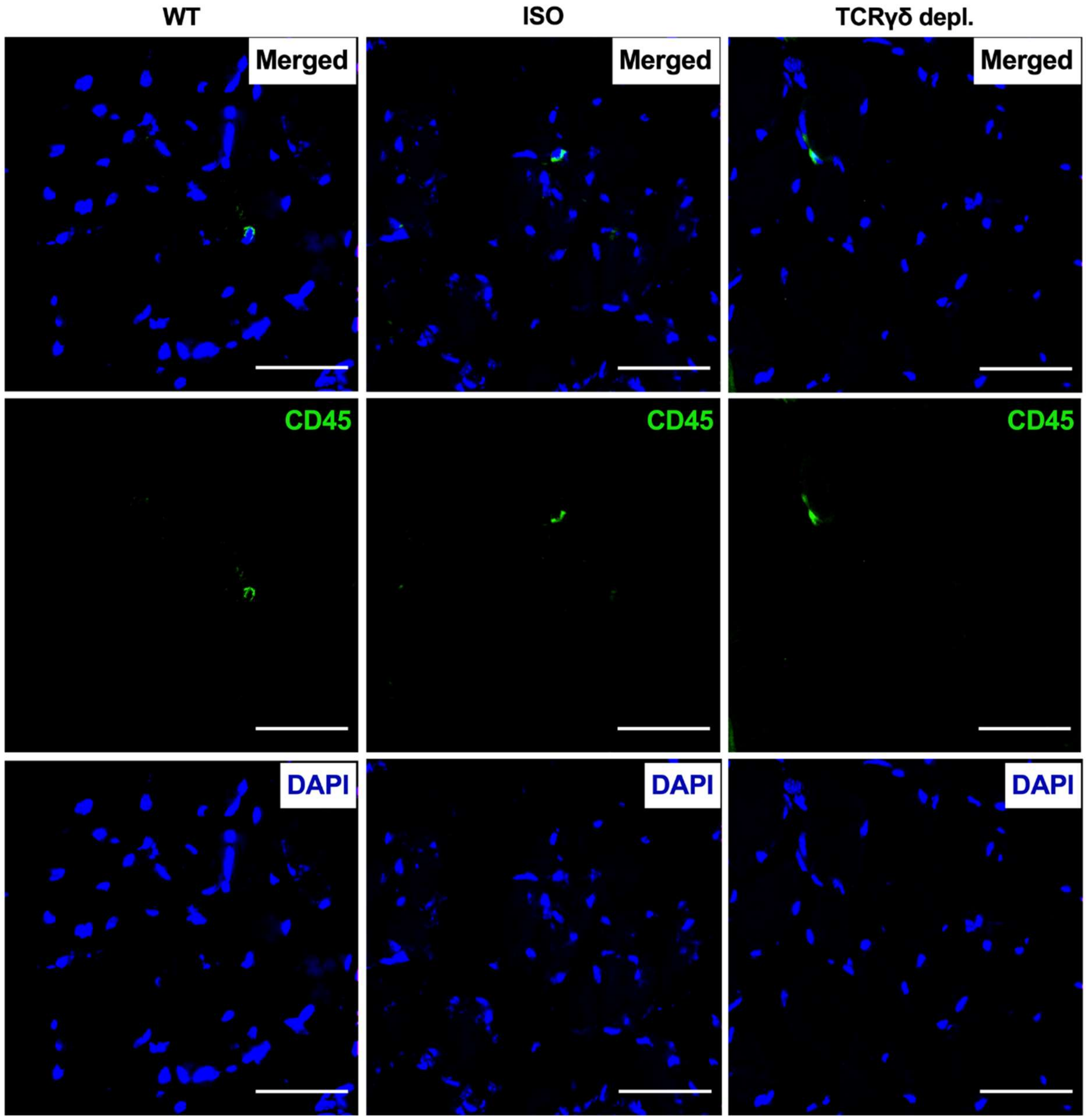

Figure S1. Wildtype (WT) (left), isotype (ISO) (middle), and γδ T cell-depleted mice (right) show low leukocyte count without any difference in number. Representative immunofluorescence images of sham-operated legs of WT (top), isotype antibody treated (middle) and TCR γδ T cell depleted mice (bottom) 7 days after surgery. Leukocytes were stained with an antibody against CD45 (green), nuclei were labeled using DAPI (blue). Scale bars: 50 µm.

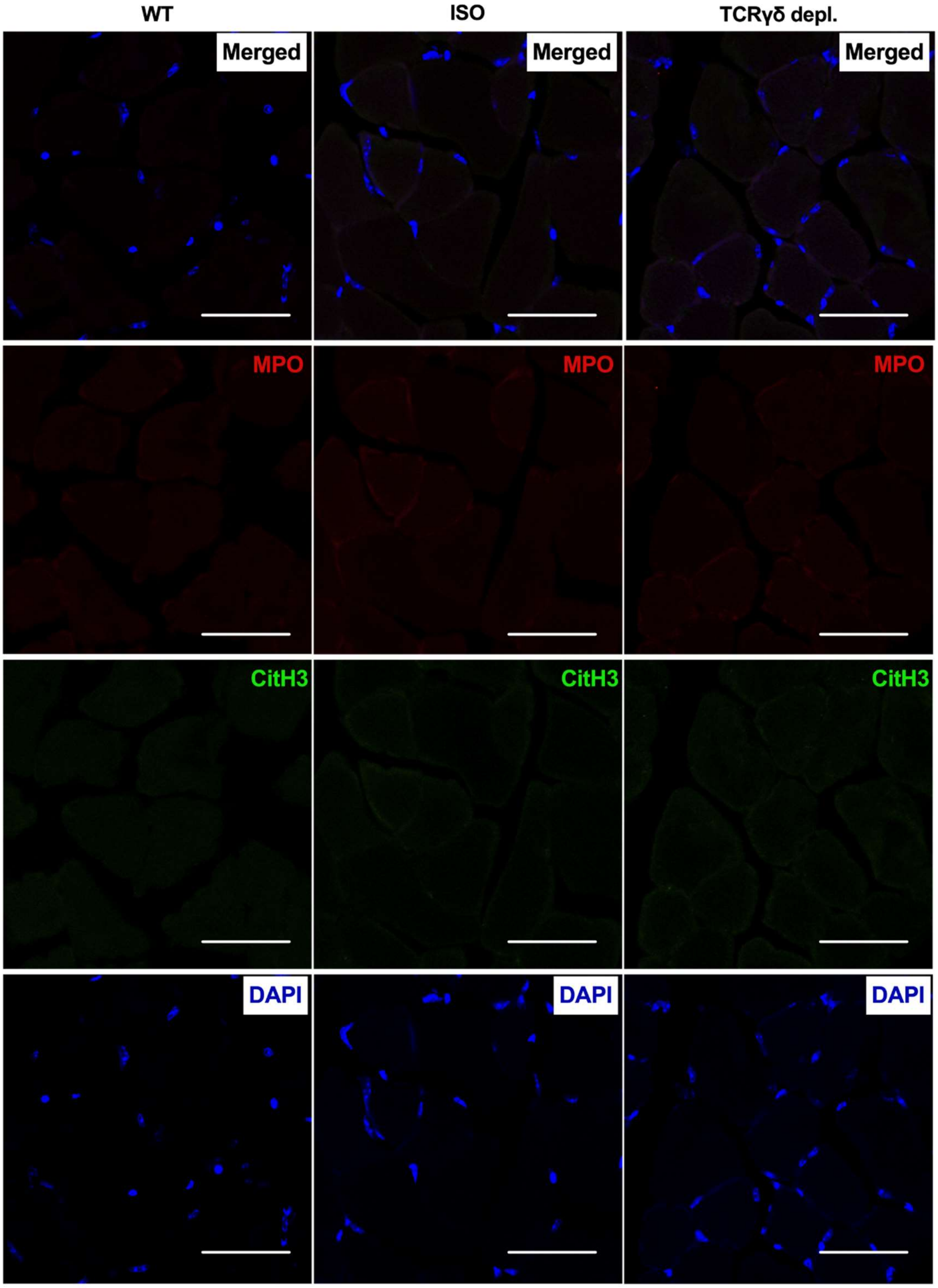

Figure S2. Representative pictures of wildtype (WT), isotype (ISO) and γδ T cell depleted mice show almost no neutrophiles or neutrophil extracellular traps (NETs). Representative immunofluorescence images of sham-operated legs of WT (left), isotype antibody treated (middle) and TCR γδ T cell depleted mice (right) 3 days after surgery. Cells were labeled with antibodies targeting MPO (marker for neutrophils, red), CitH3 (NETs, green) and DAPI (nuclei, blue). Scale bars: 40 μm.

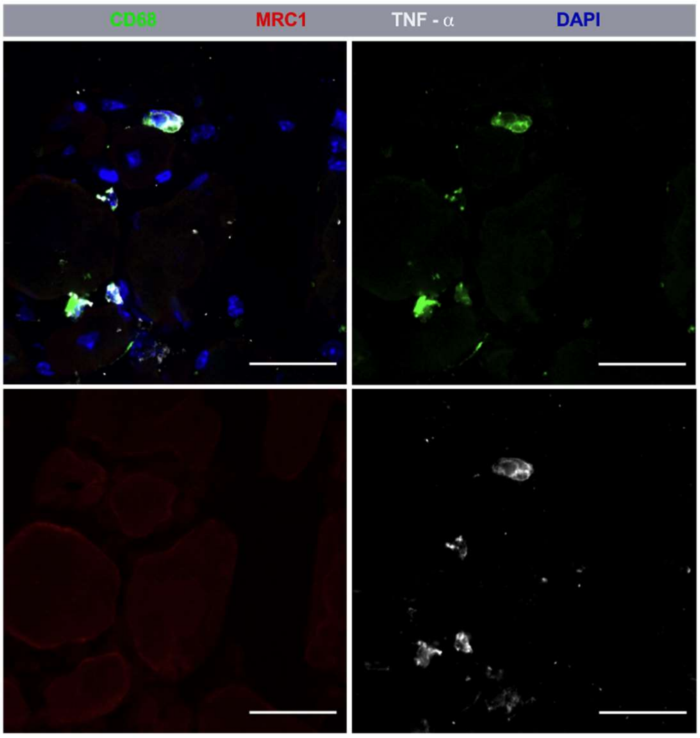

Figure S3. MRC1 negative macrophages (CD68+/MRC1-) show co-staining with TNF-α. Representative immunofluorescence image of gastrocnemius muscle of mice 7 days after femoral artery ligation. Cells were labeled with antibodies against CD68 (macrophages, green), MRC1 (mannose receptor C-type 1, red), TNF-α (tumor necrosis factor alpha, white) and DAPI (nuclei, blue). Scale bars: 30 µm.

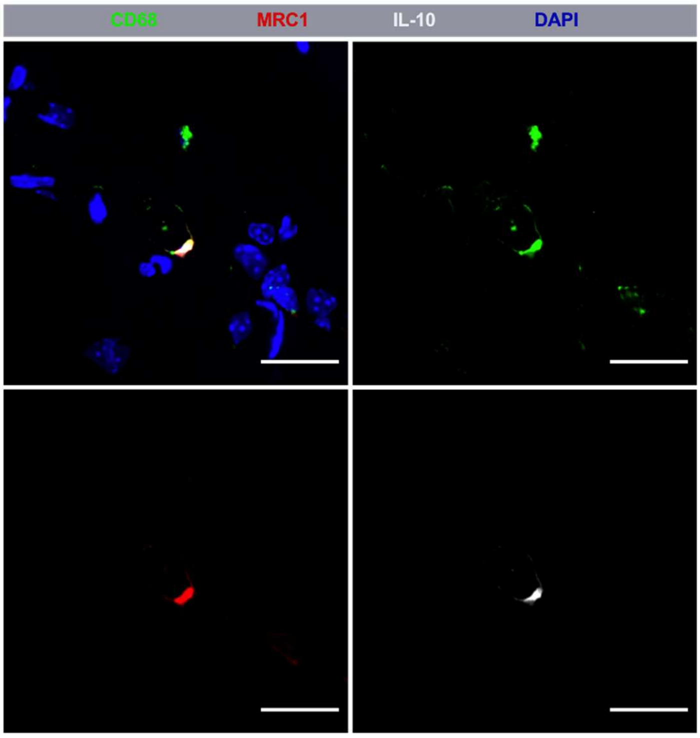

Figure S4. MRC1 positive macrophages (CD68+/MRC1+) show co-staining with IL-10. Representative immunofluorescence image of gastrocnemius muscle of mice 7 days after femoral artery ligation. Cells were labeled with antibodies against CD68 (macrophages, green), MRC1 (mannose receptor C-type 1, red), IL-10 (interleukin 10, white) and DAPI (nuclei, blue). Scale bars: 20 µm.

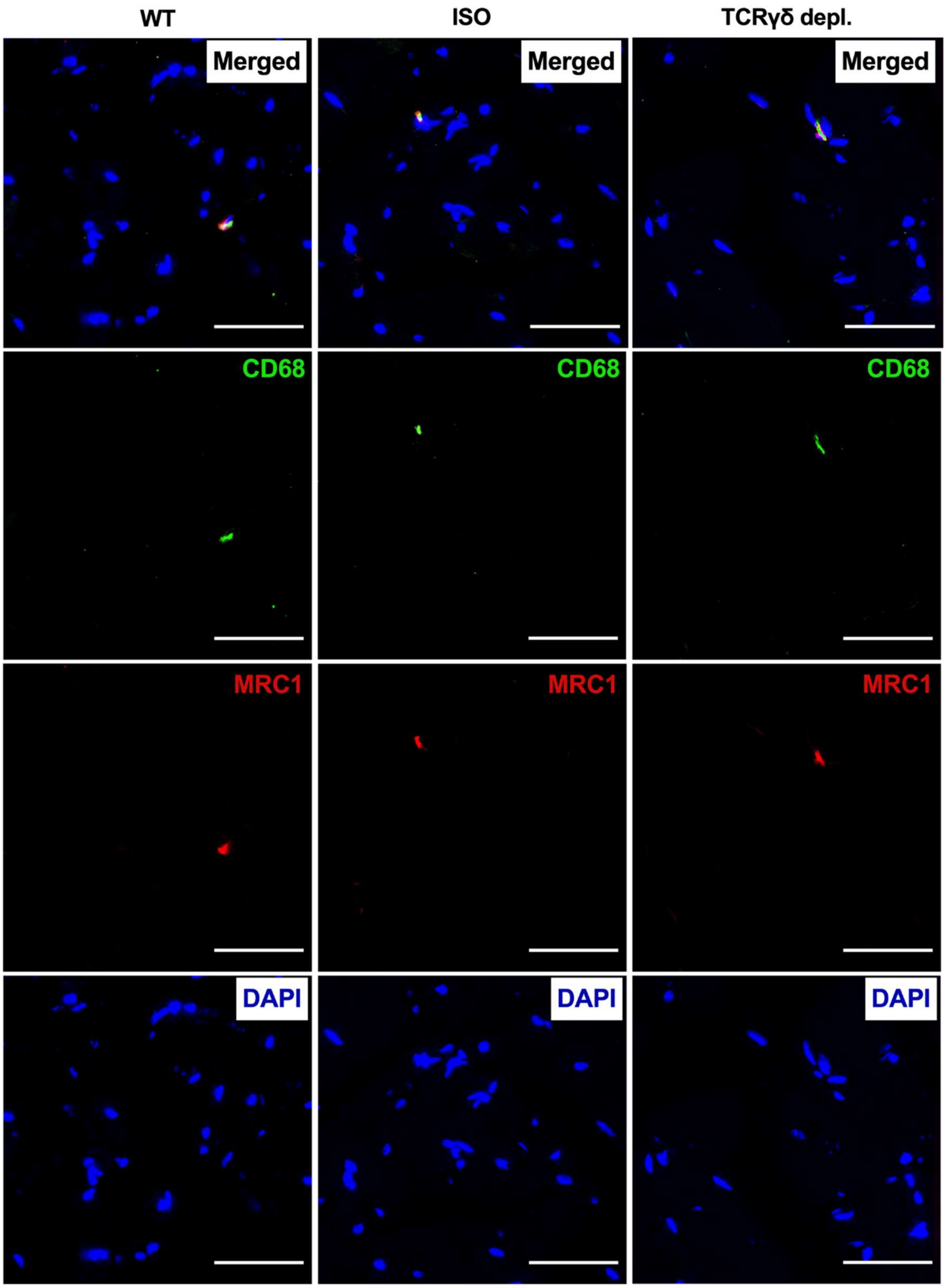

Figure S5. Wildtype (WT), isotype (ISO) and γδ T cell depleted mice show low numbers of macrophages. Representative immunofluorescence images of gastrocnemius muscles of WT (left), isotype antibody treated (middle) and TCR γδ T cell depleted mice (right) 7 days after sham-operation. Cells were labeled with antibodies against CD68 (macrophages, green), MRC1 (mannose receptor C-type 1, red) and DAPI (nuclei, blue). Scale bars: 50 μm.
